# Pediatric foreign body aspiration: How much does our community know?

**DOI:** 10.4103/0971-9261.72435

**Published:** 2010

**Authors:** Aprajita Singh, Dhruv Ghosh, Clarence Samuel, William Bhatti

**Affiliations:** Department of Paediatric Surgery, Christian Medical College and Hospital, Ludhiana, Punjab, India; 1 Department of Community Health, Christian Medical College and Hospital, Ludhiana, Punjab, India

**Keywords:** Awareness, community, foreign body aspiration

## Abstract

**Aims:**

Foreign body aspiration (FBA) is one of the main causes of accidental death in childhood. This study was designed to evaluate the level of awareness of FBA and its resultant dangers in the community.

**Materials and Methods:**

Sixty-three primary caregivers were interviewed about their awareness of FBA, its attendant dangers, preventive measures taken, and how will they take care of a child in the event of a FBA according to an agreed protocol.

**Results:**

Awareness levels about FBA were abysmally low in the population that was studied. Twenty-five percentage of the study population had not heard about this condition, and 46% could not recognize a FBA if it happened. Also, 76% of the study group did not know about the attendant dangers of this condition.

**Conclusions:**

There is a dire need to spread awareness about both prevention and treatment of this morbid condition. Health care professionals need to increase their efforts to spread more knowledge in the community about FBA.

## INTRODUCTION

Foreign body aspiration (FBA) is a frequent cause of accidental death in children below the age of 6 years all over the world.[1] It is considered a true emergency in the pediatric age group and leads up to 300 deaths per year in the USA.[2] A large number of FBAs in the tracheobronchial tree occur in the Indian sub-continent.[3,4] Educational campaigns as a public health measure in some countries have brought down the incidence of FBA as well as the associated mortality.[5,6] The aim of this study was to assess the level of awareness in parents or caregivers about FBA.

## MATERIALS AND METHODS

Primary caregivers of children who visited the Department of Paediatric Surgery at our Institution either as out-patients or in-patients formed part of this study. The primary caregivers were interviewed by the investigators in a language they are familiar with. The interview was conducted as pre a decided protocol.

Caregivers of all those children who have had an episode of FBA earlier were excluded from this study.

Using the formula *N*=*Z* × *Z*(*P*(1–*P*))/*D*× *D* where the expected prevalence of knowledge regarding FBA was 20% and the confidence level 90%, the sample size was calculated as 59. Informed consent was obtained from all the individuals that formed part of this study. Approval from the institutional research and ethics committees was also obtained.

## RESULTS

Table 1 summarizes the answers that were given by parents or primary caregivers to the questions asked by the investigators. Sixty-three primary caregivers formed part of this study with 49.2% (*n*=31) having children in the age group of 1–5 years. Parents with children less than 1 year formed the next largest group (*n*=19) and 84.1% (*n*=53) of the primary caregivers were housewives.

**Table 1:** Study summary

Median age of children (months)	24 No. (%)
Age group	
<1 year	19(30.2)
1 – 5years	31(49.2)
6 – 10years	11(17.5)
> 10years	2(3.2)
Occupation of caregivers	
Homemaker	53(84.1)
Farmer/labourer	5(7.9)
Shopkeeper/accountant	3(4.8)
Nurse/doctor	2(3.2)
Education of caregivers	
Uneducated	10(15.9)
<Class 10	9(14.3)
Intermediate	27(42.9)
Graduate	13(20.6)
Postgraduate	4(6.3)
Can a child aspirate foreign bodies?	
Yes	47(74.6)
No	16(25.4)
Recognition of foreign body aspiration	No. (%)
Yes	34(54
No	29(46)
How dangerous is foreign body aspiration?	
Not very dangerous	2(3.1)
Slightly dangerous	18(28.6)
Life threatening	34(54)
Not sure	9(14.3)
Measures to prevent foreign body aspiration	
None	38(60.3)
Educate child	4(6.3)
Small objects out of reach	17(27)
Supervise child all the time	4(6.3)
Treatment	
Treat on own	32(50.8)
Physician	29(46)
Not sure	2(3.2)
Source of information	
No information	48(76.2)
Others (relatives, friends, etc.)	6(9.5)
Visual media	5(7.9)
Allied health workers	2(3.2)
Physicians	1(1.6)
Visual media	1(1.6)

15.9% (*n*=10) of the study population was uneducated and 42.9% (*n*=27) of the primary caregivers had education at least up to class 10. Also, 74.6% (*n*=47) of the parents interviewed knew that their children could aspirate foreign bodies. Sixty percentage of the uneducated caregivers said it was not possible for children to aspirate foreign bodies. There was a statistically significant difference in the knowledge of the possibility of FBA between educated and uneducated caregivers (Fishers’s exact *P*<0.012, relative risk =0.49).

Table 2 lists the perceived symptomatology of FBA among the caregivers. Forty-six of the study population (*n*=29) said that they will not be able to recognize a FBA. Eighty percent of the uneducated parents did not know the symptomatology of FBA. Educated parents were found to be more likely to recognize a FBA when it occurred compared to uneducated caregivers and this difference was statistically significant (Fishers exact *P* value = 0.02, relative risk= 0.33). Forty-six percent of the caregivers interviewed feel that FBA is not a life-threatening event. Sixty percentages of the primary caregivers have not taken any measures to prevent FBA in their wards.

**Table 2:** Symptomatology

Symptoms	Frequency No. (%)
Cant recognize	29(46)
Breathlessness	5(7.9)
Coughing	8(12.7)
Vomiting	7(11.1)
Choking	2(3.2)
Difficulty in breathing	2(3.2)
Vomiting/breathlessness	2(3.2)
Choking/cough/vomiting	1(1.6)
Cough/dyspnoea	1(1.6)
Coughing/vomiting	1(1.6)
Difficulty in breathing/pain	1(1.6)
Dyspnoea/vomiting	1(1.6)
Haemoptysis/dyspnoea/crying	1(1.6)
Restlessness	1(1.6)
Watering of eyes/restlessness	1(1.6)
Total	63

When asked as to how they would treat the child if a FBA did occur, 50.8% (*n*=32) of the parents said that they would like to remove the foreign body on their own. Forty-six percent (*n*=29) would take the child to a general physician while 3.2% of the parents interviewed were not sure what to do if their child aspirates. Forty-nine percentage of the educated and 60% of the uneducated parents said they would remove the foreign body themselves. Education status, therefore, does not seem to significantly affect the mode of treatment (*P*=0.35). Seventy-six percent (*n*=48) of the parents interviewed had not heard about the dangers of FBA. Among those who had heard, only three had sourced their information from medical professionals. None of the uneducated parents had heard about the dangers of FBA.

## DISCUSSION

FBA remains a huge problem and a major cause of accidental death in children around the world.[7,8] The age group, 1–5 years, is most vulnerable for FBA.[1] Delays in diagnosis occur seven times more commonly in aspirations than in ingestions.[9] Delay in diagnosis can lead to serious pulmonary damage and increased risk of long-term complications.[9,10]

Breathlessness, excessive coughing, and vomiting were considered the main symptoms of FBA by our study population, similar to the “Penetration Syndrome” described by Koul *et al*.[11] Similarly in a study by Kirtane *et al*., the common symptomatology spectrum reported is similar to what was suggested by caregivers who were included in our study.[8] It, therefore, appears that parents who do know about FBA have a fair idea about the symptomatology that comes along with it. Unfortunately, only half of the parents we interviewed were confident that they would be able to recognize an episode of FBA which probably would cause delay in diagnosis. According to our study, caregivers who have had some kind of education are five times more likely to recognize a FBA than uneducated caregivers (χ^2^=5.52).

While morbidity in FBA has been associated with diagnostic delay, it has also been associated with delay in getting expert medical help.[2,8,12] Half of the primary caregivers (51%) in our study said that they would try to remove the foreign body themselves, probably leading to a possible delay in instituting correct management.

Information about FBA and its attendant danger in our community seems to be abysmally low. If we consider our study population as a sample of the community in a large urban industrial city like Ludhiana, then only 23.8% have some information about FBAs. Our study found that uneducated caregivers had a 7.5 times higher chance of having no knowledge of this entity than educated ones (χ^2^=7.51). At the same time almost half the parents interviewed thought that FBA is not a life-threatening situation. Keeping small objects out of the reach of children and education of older children appears to be the most popular way of preventing FBA[13] Results in our study reflect an ambivalent attitude toward this life-threatening condition with 61.3% of the caregivers in the high-risk group (1–5 years) having taken no measures in their homes to prevent FBA.

Increase in awareness of FBA among the community has shown to decrease the morbidity and mortality as well as the incidence of this disease.[6,14] An educational campaign in Israel showed a decrease in the number of cases of FBAs during the period 2000–2004 compared to 1991–1999. The decline in the total number of bronchoscopies was thought to be due to a result of a campaign for proper education of public and especially parents, caretakers, and families.[13,14] The risk of high morbidity and mortality from FBA makes it mandatory to increase even more the awareness of the general population. As mentioned earlier 76% of the parents interviewed had not heard about the dangers of FBA. Only 3 out of the 15 parents who had heard about the dangers of FBA had sourced their information from medical professionals. It seems as medical professionals we have fallen woefully short of our role in dispensing important and relevant information regarding the dangers of FBA and measures to prevent it.

FBA is largely preventable, and the morbidity and mortality associated with it can be significantly reduced by educating the public regarding its potential dangers. Most importantly, physicians and health workers can play a significant role in spreading awareness about FBA in the community. Sensitizing the community toward the need of awareness regarding FBA through educational campaigns involving mass media and stricter measures if taken by the primary care givers can drastically reduce the morbidity and mortality associated with FBA.

Educational campaigns carried out through television and radio broadcasts, articles, and interviews in newspapers especially vernacular newspapers and educational programs at pediatric outpatient departments and wards using audio visual aids can increase community awareness about the dangers of FBA.[6] Well baby cards and immunization records which are widely distributed in India should have the dangers of FBA printed prominently on them. Similarly caregivers should be educated about the dangers of foreign body inhalation in well baby clinics. Awareness should be inculcated in *angan wadi* workers, trained birth assistants, family physicians, and pediatricians to tell parents about FBA while introducing weaning diets as food items especially nuts have been shown to be commonly aspirated in the Indian scenario.[8] Finally, we also suggest that there should be stricter guidelines for toy manufacturers as well as prominent product safety labelling that will inform consumers of the dangers of choking through age appropriate labelling of toys and high-risk items which can be aspirated.[14]
